# Therapeutic Significance of Natural Anti Oxidants

**DOI:** 10.17795/jjnpp-13614

**Published:** 2013-07-20

**Authors:** Ali Asghar Hemmati

**Affiliations:** 1 School of Pharmacy, Ahvaz Jundishapour University of Medical Sciences, Ahvaz, IR Iran

**Keywords:** Free Radicals, Antioxidants, Therapeutics, Flavonoids

It is obvious that the life of many animal spices require oxygen. However, oxygen is a highly reactive molecule that damages living organisms by producing reactive oxygen radicals. The reactive oxygen species produced in cells include hydrogen peroxide (H_2_O_2_), hypochlorous acid (HClO), and free radicals such as the hydroxyl radical (•OH) and the superoxide anion (O_2_−).This species is produced from hydrogen peroxide in metal-catalyzed redox reactions such as the Fenton reaction. These oxidants can damage cells by starting chemical chain reactions such as lipid peroxidation, or by oxidizing DNA or proteins (1). DNA damage can cause mutations and possibly cancer, if not reversed by DNA repair mechanisms, while damage to proteins causes enzyme inhibition, denaturation and protein degradation (1). On the other hand, living organisms contain a complex network of antioxidant metabolites and enzymes that aim to abolish oxidative damage to cellular components such as DNA, proteins, and lipids. In general, antioxidant systems either prevent these reactive species from being formed, or remove them before they can damage vital components of the cell. However, reactive oxygen species also have useful cellular functions, such as redox signaling. Thus, the function of antioxidant systems is not to remove oxidants entirely, but instead to keep them at an optimum level (2). In recent years, the role of oxidative stress has been postulated in many conditions, including atherosclerosis, inflammatory conditions, certain cancers, and the process of aging. In many cases, this follows the observation of increased amounts of free radical damage products, particularly markers of lipid peroxidation, in body fluids (3). Fortunately different groups of antioxidants are available, which can be used for prophylaxis or therapeutically in diseases related oxidative stress. Apart from classic antioxidants, e.g. Vit E and Vit C we have access to a large numbers of antioxidants with natural origin. Among them flavonoids are a group of polyphenolic antioxidants found in many fruits, vegetables, and beverages such as tea. Over 4000 flavonoids have been identified such as flavonols (quercetin and kaempherol), flavanols (the catechins), flavones (apigenin), and isoflavones (genistein). Several studies suggest an inverse relation between flavonoid intake and incidence of chronic diseases such as coronary heart disease (4). Existence of such a valuable source of natural drugs which can use in a wide range of diseases is an opportunity for medical research in order to get the best benefits of them for prevention and treatment of diseases. 

## References

[1] Valko M, Leibfritz D, Moncol J, Cronin MT, Mazur M, Telser J (2007). Free radicals and antioxidants in normal physiological functions and human disease.. Int J Biochem Cell Biol..

[2] Knight JA (1998). Free radicals: their history and current status in aging and disease.. Ann Clin Lab Sci..

[3] Heinecke JW (1997). Mechanisms of oxidative damage of low density lipoprotein in human atherosclerosis.. Curr Opin Lipidol..

[4] Young IS, Woodside JV (2001). Antioxidants in health and disease.. J Clin Pathol..

